# Relationship Between Group Cohesion and Anxiety in Soccer

**DOI:** 10.2478/v10078-012-0071-z

**Published:** 2012-10-23

**Authors:** Carla Chicau Borrego, Luis Cid, Carlos Silva

**Affiliations:** 1 Sport Sciences School of Rio Maior - Polytechnic Institute of Santarém. Research Center in Sports, Health Sciences and Human Development - CIDESD.

**Keywords:** Group Dynamics, cohesion, anxiety, soccer

## Abstract

Group cohesion in sport is a widely spread theme today. Research has found cohesion to be influenced by several individual and group components. Among the cognitive variables that relate to cohesion we found competitive anxiety. The purpose of this study was to examine the relation between task cohesion (ATG-T, and GI-T) and competitive state anxiety (A-state), and also if there would be a relation between cohesion and self-confidence. Participants were 366 football players of both genders male and female, aged between 15 to 23 years old, from Portugal’s championships. Cohesion was measured using the Portuguese version of the Group Environment Questionnaire, and to assess competitive anxiety, we used the Portuguese version of the Competition State Anxiety Inventory 2. Our results show that female athletes report experiencing more cognitive anxiety and less self-confidence than male athletes. Only cognitive anxiety relates in a significantly negative way with the perception of cohesion (GI-T e ATG-T) in the total number of participants and in male athletes. Relatively to the somatic anxiety, it only relates negatively with the perception of the integration of the group in the total number of participants and in the male gender.

## Introduction

The concept of group cohesion has been an ongoing interest of social psychologists for a long time. Sport psychologists have also contributed to this, and the works of Carron et al. (1985; 1998) are well known and recognized at the international level. Carron (1982) defines group cohesion as “the tendency for a group to stick together and remain united in the pursuit of its instrumental objectives and/or for the satisfaction of member affective needs”, as a multidimensional construct comprised of both task and social aspects, and involving the dual processes of integration into the group and attraction toward other group members (Carron et al., 1985; Carron et al., 1998). Thus, four dimensions come into sight: Individual Attractions to the Group-Social (ATG-S), Individual Attractions to the Group-Task (ATG-T), Group Integration-Social (GI-S) and Group Integration-Task (GI-T).

In a reflexive analysis of the work developed by Carron et al. (1985), Cota et al. (1995), outlined the multidimensional model which based on the two fundamental reasons: “First, both of the presented dimensions are very important to understand cohesion in different types of group, having been identified by other authors in an independent form. Second, the implications of both subscales were tested in a growing number of works”. Also Blanchard et al. (2000) suggest the relevance of the GEQ as the most frequently chosen instrument among sport psychologists to access group cohesion.

The perception of high levels of cohesion is highly related to the sensation of the group unit, the collective and interdependence with the team members, while the perception of low levels of cohesion is related to the sensation of individual orientation, the nonexistence of cooperation and independence of the team members (Carron et al., 1998).

Research has found cohesion to be influenced by several individual and group components. A relationship was found between cohesion and such components as: satisfaction (Aoyagi et al., 2008; Spink et al., 2005) performance (Carron et al., 2002), role ambiguity (Beauchamp et al., 2003), mood (Terry et al., 2000) and cognitive variables as competitive anxiety (Prapavessis and Carron, 1996; Cogan and Petrie, 1995).

Anxiety is a negative emotion that affects perceptions in sport competitions, and this leads the majority of athletes to consider anxiety as debilitative towards performance, which may result in a decrease in performance (Raglin and Hanin, 2000; Weinberg and Gould, 1999). Martens et al. (1990) developed the multidimensional model of anxiety where a distinction on reactions of anxiety in sport is presented, “cognitive anxiety is usually defined as the mental component of anxiety and is caused by negative expectations” while somatic anxiety “refers to the physiological and affective elements of the anxiety experience that develop directly from autonomic arousal”. A third dimension related with the above two is an individual difference factor, which is self-confidence, understood as the conviction of the athlete that he can perform the tasks which he has undertaken. Cognitive anxiety and self-confidence represent the opposite ends of a continuous cognitive assessment. Martens et al. (1990) propose a negative linear relationship between cognitive anxiety and performance, and a positive linear relationship between self-confidence and performance. Somatic anxiety and performance have a curvilinear relationship, where both lower and higher values are prejudicial to performance.

Researchers have continued to examine issues related to the multidimensional anxiety theory, and involved the examination of potential interactive effects between sport competitive anxiety and other components. The relationship between group cohesion and competitive state anxiety appears to be a dynamic one in which both variables influence each other (Eys et al., 2003). This also speaks to the degree of team cohesion. That is “improving the dynamics of the team could enhance the psychological state of the individual” (Prapavessis and Carron, 1996). Additionally, Cogan and Petrie (1995) found that an intervention program with intercollegiate gymnasts was associated with enhanced social cohesion and reduced somatic and cognitive anxiety. Also, a significant number of the athletes who required consultation were those who were suffering from anxiety, before and during competitions (Bull, 2000).

Prapavessis and Carron’s (1996) findings revealed that cohesion and anxiety were associated. Particularly, athletes that perceived higher levels of task cohesion reported a state of less cognitive anxiety. Results also evidence that psychological costs associated with membership on cohesive teams, mediates the cohesion - state anxiety relationship.

However, benefits of group cohesion go beyond the degree of competitive state-anxiety. Eys et al. (2003) indicate that participating in a cohesive group leads to higher self-esteem, increased group-efficacy, better mood and higher dissemination of responsibility among group members. Additionally, individuals who participate in a group sport are less likely to experience competitive state-anxiety in general (Craft et al., 2003). Courneya (1995) provided additional support for a cohesion-affect link by showing that perceptions of group cohesion were associated with positive feelings towards structured exercise classes.

As Martin and Hall (1997) refer, it is unclear whether this difference between sports is due to the sports themselves or due to the sports attracting individuals with different characteristics. The outcome of the competitive state anxiety also depends on the type of skill (i.e., open or closed). Open skills have been defined by Craft et al. (2003) as those skills in which the athlete is “performing in an interactive ever-changing environment”, represented for example by soccer, and are more likely to be more influenced by competitive state anxiety than closed skills (Terry and Youngs, 1996). Another personal characteristic is gender, as female athletes report higher cognitive anxiety and lower self-confidence than males (Martens et al., 1990; Wark and Wittig, 1979; Jones et al., 1991; Cartoni et al., 2005). Therefore, this is also a preliminary contribute to distinguish the anxiety level between genders.

Task cohesion variables were the only ones used for three reasons. First, following the suggestion of Munroe et al. (1999), athletes tend to be involved in sport competition due to instrumental or tasks objectives. Second, Prapavessis and Carron (1996) only found a significant relationship between pre-competitive anxiety and the individual attraction to group dimension, while for Eyes et al. (2003) the relationship with anxiety was manifested in both dimensions of the task cohesion. The third reason was presented by Prapavessis and Carron (1996) when in their results they suggested that athletes with higher perception of cohesion tended to indicate that pressure associated to responsibilities and satisfaction of the needs of others (i.e. task orientated activities) was more reduced, thus they experienced less anxiety. The inclusion of the third subscale was due to the fact that Craft et al.’s (2003) meta-analysis demonstrates that self-confidence seems to be the strongest indicator of sport performance compared to the remaining subscales of anxiety (somatic and cognitive), assessed by CSAI-2. Also Moritz et al. (2000) identified self-efficiency as a predictor of sport performance.

The purpose of this study was to examine the relationship between task cohesion (ATG-T, and GI-T) and competitive state anxiety (A-state). Also if there would be a negative relationship between cohesion-competition A-state (cognitive and somatic) and a positive one between cohesion-self-confidence. Additionally, we hypothesized that females perceive more cognitive and somatic anxiety than males.

## Methods

### Participants and Measures

A total of 366 soccer players, who took part in Portugal’s championships, participated in the study, with the average age of 17.14 ± 1.6 years. From the total number of the participants, 322 were males and 44 were females. They all had three structured training sessions (+/− 1,30 h) per week and one match during the weekend. All participants or parents received an information letter and signed a written consent approved by the School review board.

*Task* Cohesion. Cohesion was measured by using the Portuguese version of *Group Environment Questionnaire*, (GEQp, Cruz and Antunes, 1997). The GEQ is a 18 item questionnaire that assesses four dimensions of cohesion: Individual Attractions to Group: Social -ATG-S (e.g. “Some of my best friends are in this team”); and Task - ATG-T (e.g. “I don’t like the style of play in this team”); Group Integration – Social - GI-S (e.g. “Our team would like to spend time together in the off season”) and Task- GI-T (e.g. “Our team is united trying to reach its performance goals”). Participants answered in a 9-point Likert scale, ranging from 1 (*strongly disagree*) to 9 (*strongly agree*). As mentioned before, for this study only the two task oriented scales were used (Group integration – task – which measured the individual’s perception of the degree of unity in the team as a collective around its goals and objectives; Individual Attractions to Group – Task - which measured the individual’s perception of his/her own involvement in task oriented aspects of the group). Thus higher scores reflect higher perceptions of cohesion. In terms of internal consistency we found adequate values for both subscales: ATG-T, α= .65; GI-T, α= .75.

*Pré-competition Anxiety:* To assess competitive anxiety, we used the Portuguese version of the Competition State Anxiety Inventory 2 (CSAI-2p: Serpa and Santos, 1991), which consists of 27 items, grouped in three subscales: cognitive anxiety (CA; e.g. “I am concerned about performing poorly”), somatic anxiety (SA; e.g. “I feel nervous” or “I feel jittery”) and self-confidence (SC; e.g. “I feel self-confident”). Each subscale has items scored in a 4-point scale (from “not at all – 1” to “very much – 4”). A good internal consistency was found in this subscales, with CA (α= .87), SA (α= .80) and SC (α= .87).

### Procedures

Data was collected after explaining the goals to the head coaches of these teams and after obtaining legal authorization (parents and coaches). Athletes were briefed in the locker rooms, about the nature of the study and all concepts were clarified beyond any doubts before completing the questionnaire. The CSAI-2 was collected +/− 60 minutes before the game, in line with suggestions from Craft et al. (2003) and with no changes in the team’s routine. Confidentiality was also guaranteed.

### Analysis

Data was analyzed by means of descriptive statistics to characterize participants (mean and SD) and screened for normality with the Kolmogorov-Smirnov test. In order to analyze the relation between concepts, we used the Pearson test. Stepwise multiple regressions were used to verify main anxiety predictors.

All analysis were processed using Statistical Package for Social Sciences (SPSS for Windows, version 17.0). A significance level of 5 % was adopted for the study.

## Results

Taking into account the fact that previous research presented significant differences in pre-competitive anxiety levels (Krane and Williams, 1994; Jones and Cale, 1989), we conducted a MANOVA test with 2 (male, female) x 5 (2 task cohesion, 3 pre-competitive anxiety).

The effect of gender influence revealed statistically significant differences, with Wilks’ lambda = .78, (F _5,360_ = 20.81; p = 0.001), with a global effect size of .22 ( 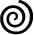
^2^ partial), corresponding to 22%. However, results of a univariate statistics analysis (Table 1) should be treated with some caution, as, although significant differences were found between genders at pre-competitive anxiety dimensions (p < 0.01), no difference was registered in task cohesion (ATG-T e GI-T). Therefore, independent analysis was adopted for females and males, and also for the total number of participants. Nevertheless, it should be highlighted that an important limitation of this study is a small number of female soccer players what means that caution must be exercised in the discussion of the results.

Through the descriptive analysis, we observed that females present higher mean values in individual attraction to the group and lower mean values in group integration, both in reference to the task, in comparison with the male gender, although these differences are not significant. As suggested in previous studies (Jones and Cale, 1989), females reveal significantly higher mean values (p < 0.01) at the cognitive anxiety level before competition than males. However, males reported experiencing significantly higher self-confidence than females (p < 0.01). Somatic anxiety presents values near significance (p = 0.069), being higher in women.

The mean differences between individual attraction (ATG-T) and integration in the group (GI-T) associated to the task, as well as between somatic (ASom) and cognitive (ACog) anxiety for overall participants, and both male and female genders, revealed to be significant (p < 0.01). In other words, males as well as females perceive themselves to be more attracted to the group rather than integrated in it, as far as task is concerned, and report experiencing more cognitive anxiety than somatic, before competition.

When we consider the total number of participants, we can verify (Table 2) that task integration in the group (GI-T) is significantly correlated (p < 0.01) to: a) self-confidence - low but positively; b) somatic - low negatively and, c) cognitive anxiety - moderate negative. Individual attraction to the group is significantly related in a low negative way to cognitive anxiety.

For female subjects, the bivariate correlation revealed that task cohesion does not correlate (p > 0.05) with the dimensions of pre-competitive anxiety, except for group task integration and self confidence (p < 0.01). For male subjects, individual attraction to the group, as well as integration in the group, both in relation to the task, are: a) positively but low related (p < 0.01) to self-confidence, and b) negatively, low to moderate correlated (p < 0.01), to cognitive anxiety. A negative but low correlation is also observed between somatic anxiety and group task integration.

### Relationship between Task Cohesion and Cognitive Anxiety

To analyze the effect between task cohesion and cognitive anxiety, measured through the dimensions of individual attraction and integration in the group (task related), regression analyses were computed, separately according to gender and the total number of participants. As stated previously, task cohesion is negatively related with cognitive anxiety. In order to maintain the degrees of freedom, only previously correlated dimensions of cohesion with cognitive anxiety were used in the stepwise regression equation (Cohen, 1988).

For the total number of participants, integration in the group associated to the task (GIT) explains 19.4% of the variance (F_1.364_ =87.74; p < .001), and the individual attraction to the group associated to the task explains only 0.6% of the variance, (F_1,364_ = 26.64, p < 0.001). Both negatively predicted cognitive anxiety (standardized β = −.44; p < 0.001 and standardized β = −.26; p < 0.001, respectively). When the regression analysis was restricted to the male gender, we verified that group integration explained 22% of the variance, (F_1.320_ = 90.06; p < 0.001) and negatively predicted cognitive anxiety (standardized β = −.47; p < 0.001), while individual attraction totaled 0.8% of the explained variance (F_1.320_ = 30.17; p < 0.001) and negatively predicted cognitive anxiety (standardized β = −.29; p < 0.001). In a previous analysis of the female gender, we found no correlations of any kind between cognitive anxiety and the cohesion variables. Therefore, no regression was carried out.

### Relationship between Task Cohesion and Somatic Anxiety

For the analysis of the relationship between individual attraction and group integration associated to the task and somatic anxiety, we used regression analysis following the same procedures as previously described. For females, once again no correlations between somatic anxiety and the dimensions of the task cohesion were found, thus regression was not carried out. A similar situation was verified for the total number of participants and also for the male gender, in the variable of individual attraction to the group. We then continued with the analysis of the regression model, in which the variable of integration in the group, the only predictor of somatic anxiety, explained for the total of participants variance at the level of 0.7%, (F_1.320_ = 27.85; p < 0.001) and negatively predicted somatic anxiety (standardized β = −.27; p < 0.001), and for the male gender, variance at the levele of 0.8%, (F_1.320_ = 28.07; p < 0.001) and negatively predicted somatic anxiety (standardized β = −.28; p < 0.001).

### Relationship between Task Cohesion and Self-Confidence

Identical procedures were followed in the analysis of the effect between task cohesion and self-confidence implementing one stepwise regression analysis separately according to gender and also to the total number of participants. For the overall sample, integration in the group associated to the task (GI-T) explained variance of 0.4%, (F_1.364_ =14.4; p < 0.001) and positively predicted self-confidence (standardized β = .19; p < 0.001). Individual attraction to the group associated to the task explained only variance of 0.1%, (F_1.364_ = 26.64, p < 0.001). When the analysis of regression was restricted to the male gender, we verified that integration in the group explained variance of 0.4%, (F_1.320_ =12.19; p < 0.001), where the dimension individual attraction totaled 0.2% of the explained variance, (F_1,320_ =6.71; p < 0.001); both positively predicted self-confidence (standardized β = .19; p < 0.001 and standardized β = .43; p < .001, respectively). Once again in the previous analysis, no correlation between self-confidence and the studied variables of cohesion were verified in the female gender, thus no regression was carried out.

## Discussion

The aim of this study was to analyze the relationship between cohesion and the pre-competitive state of anxiety, specifically the association between the perception of integration and individual attraction to the group associated to the task, the perception of somatic and cognitive anxiety, as well as self-confidence before competition. Results of the present study (due to the number of female athletes) show that athletes of the female gender report experiencing more cognitive anxiety and less self-confidence than athletes of the male gender. Cognitive anxiety relates in a significantly but low negative way with the perception of cohesion (GI-T and ATG-T) in the total number of participants and in the male gender. With regard to somatic anxiety, it only relates negatively to the perception of integration in the group in the total number of participants and in the male gender. The best predictor of cognitive anxiety was GI-T, being the only one in case of somatic anxiety. No relationship was found between task cohesion and competitive anxiety in the female gender.

Our results are partially consistent with the theory presented by Baumeister and Leary (1995), in which the feeling of belonging is inversely associated with the negative aspects and directly with positive aspects, which means that, for the male gender, more cohesive groups tend to experience less anxiety and more self-confidence before competition. However, individual attraction to the group did not present any correlation with pre-competitive somatic anxiety. This result was surprising, as this association ATG-T-somatic anxiety was present in the study of Prapavessis and Carron (1996). The results that indicate that females report experiencing more cognitive anxiety compared to males, partially confirm the results of Martens et al. (1990), Jones et al. (1991), and Cartoni et al. (2005) as it has been stated that males present higher levels of self-confidence, compared to females.

As referred to previous research, the best predictor for both, cognitive and somatic anxiety was integration in the group associated to the task, confirming the relationship of cognitive factors associated with cohesion. This relation is supported by two studies that highlight this fact. First, Prapavessis and Carron (1996) found that athletes who had higher perceptions of cohesion experienced less cognitive anxiety. Second, Eys et al. (2003) extended the Prapavessis and Carron’s (1996) study and found that athletes who interpreted their symptoms of anxiety as facilitative to their performance were also more likely to perceive higher team cohesion. Nevertheless, this relationship should be analyzed with precaution since both cohesion as well as cognitive anxiety present associations with other components, where the influence of one upon the other could depend on other factors such as the perception of difficulty versus facility of a competition and possible outcomes, coping strategies, self-confidence, athlete satisfaction and prior experiences. Furthermore, Terry et al. (2000) documented that belonging to a cohesive group improves one’s state of mind.

In conclusion, female athletes report less self-confidence than male athletes and this may be a reason why they report experiencing more cognitive anxiety. On the other hand, the significantly negative correlation between cognitive anxiety and the perception of cohesion (GI-T and ATG-T) in males, supports the statement that cohesive groups tend to deal better with anxiogenic situations. Relatively to the somatic anxiety, it also only relates negatively with the perception of the integration of the group in males, supporting the previous conclusion. All behaviors associated with individual attraction towards the task are increasing factors of anxiety, since the best predictor of cognitive anxiety were GI-T, being the only one in case of somatic anxiety. No relationship was found between task cohesion and competitive anxiety in females.

The present study has some limitations that need to be taken into account when considering its contributions. Since the level of anxiety after the competition is not related to the athletes’ performance, this study merely focused on anxiety levels before the competition. The heterogeneous nature of the sample (i.e. male and female players) and the limited number of female soccer players mean that caution must be exercised before generalizing the results to other populations. Future research might examine the relationship between cohesion and athletes’ affective states within teams from different sports disciplines (i.e. open sport versus closed sport), competitive levels (Radochoński et al., 2011) and enlarge female samples. Also, recent research by Jones and colleagues (Jones et al., 1993; Jones and Hanton, 2001; Jones et al., 1994; Jones and Swain, 1992) supports the suggestion that although scores on the CSAI-2 reflect the intensity of anxiety symptoms, they provide no insight into how athletes interpret those symptoms, suggesting further research focusing on Jones’ (1991; 1995) directionality hypothesis within the context of the Multidimensional Anxiety Theory (Martens et al., 1990), in Portuguese athletes.

An example of the practical implications of results from the present study is that coaches, sport psychologists, and counselors can make use of the reported findings to provide appropriated strategies in group sport levels and athletes who showed the highest level of anxiety in order to reduce their anxiety level before and during the competition.

## Figures and Tables

**Table 1:** Descriptive Statistics for Cohesion and Anxiety dimensions

	Total		Male		Female	
	Mín-Max	M (SD)	Mín-Max	M (SD)	Mín-Max	M (SD)

ATG-T	1–9	6.96 (1.71)	1–9	6.94 (1.78)	4–9	7.11 (1.20)
GI-T	2–9	6.43 (1.37)	2–9	6.45 (1.44)	3.80–8	6.30 (0.85)
SomA	1–4	1.81 (0.53)	1–4	1.69 (0.63)	1.11–2.44	1.71 (0.37)
CogA	1–4	1.96 (0.68)	1–4	1.90 (0.69)	1.33–3.33	2.38 (0.48)**
SelfConf	1–4	3.09 (0.59)	1–4	3.16 (0.57)	1.56–3.67	2.58 (0.58)**

N = 366 (322 males. 44 females)

* p < 0.05.

** p < 0.01

**Table 2:** Correlation coefficients between task cohesion and competitive anxiety

	ATG-T	GI-T	ASom	ACog	AutoConf
ATG-T					
GI-T	.475**				
SomA	−.085	−.257**			
CogA	−.261**	−.441**	.623**		
SelfConf	.101	.195**	−.322**	−.367**	

Correlations total sample, respectively

* p < 0.05;

** p < 0.01

**Table 3:** Task cohesion and competitive anxiety correlations in males and females

	Male				Female			
	ATG-T	GI-T	ASom	ACog	ATG-T	GI-T	ASom	ACog
ATG-T								
GI-T	.503**				.003			
SomA	−.093	−.284**			.082	−.009		
CogA	−.294**	−.469**	.674**		.008	−.012	.245	
SelfConf	.143**	.192**	−.361**	−.306**	−.177	.405**	−.406**	−.492**

Correlations in males (n = 322) and females (n =44), respectively

* p < 0.05;

** p < 0.01
